# Investigation on Selective Laser Melting AlSi10Mg Cellular Lattice Strut: Molten Pool Morphology, Surface Roughness and Dimensional Accuracy

**DOI:** 10.3390/ma11030392

**Published:** 2018-03-07

**Authors:** Xuesong Han, Haihong Zhu, Xiaojia Nie, Guoqing Wang, Xiaoyan Zeng

**Affiliations:** 1 Wuhan National Laboratory for Optoelectronics, Huazhong University of Science and Technology, Wuhan 430074, China; hanxuesong@hust.edu.cn (X.H.); xjnie@hust.edu.cn (X.N.); xyzeng@hust.edu.cn (X.Z.); 2 China Academy of Launch Vehicle Technology, Beijing 100076, China; wanggq15@126.com

**Keywords:** selective laser melting, cellular lattice structure strut, molten pool morphology, surface roughness, powder-supported zone

## Abstract

AlSi10Mg inclined struts with angle of 45° were fabricated by selective laser melting (SLM) using different scanning speed and hatch spacing to gain insight into the evolution of the molten pool morphology, surface roughness, and dimensional accuracy. The results show that the average width and depth of the molten pool, the lower surface roughness and dimensional deviation decrease with the increase of scanning speed and hatch spacing. The upper surface roughness is found to be almost constant under different processing parameters. The width and depth of the molten pool on powder-supported zone are larger than that of the molten pool on the solid-supported zone, while the width changes more significantly than that of depth. However, if the scanning speed is high enough, the width and depth of the molten pool and the lower surface roughness almost keep constant as the density is still high. Therefore, high dimensional accuracy and density as well as good surface quality can be achieved simultaneously by using high scanning speed during SLMed cellular lattice strut.

## 1. Introduction

Metal cellular lattice structures are a unique class of structures with combinational advantages, such as: low densities, superior mechanical, thermal, electrical, and acoustic insulation properties. However, conventional production techniques confront high difficulty to fabricate such kinds of complex structures.

Selective laser melting (SLM), as one of powder bed based additive manufacturing techniques, can produce near-net-shaped parts with customized and complicated structure directly from computer-aided design data [1,2,3,4,5,6]. So, it is very suitable to make metal cellular lattice structures beyond current limitations. In fact, SLM technology has attracted much attentions to fabricate cellular lattice in recent years [7,8,9,10].

For a specific cellular lattice structure, the inside inclined struts play a central role in determining its performance, such as elastic modulus, tensile strength, yield strength, etc. During SLM, the struts generally suffer poor surface quality and low dimensional accuracy as well as internal defect. Poor surface quality is very difficult to finish and leads to early fracture and low mechanical properties [11,12]. Large geometrical error induces weight and mechanical properties deviating from the target value [13,14]. The internal defects deteriorate the mechanical properties. Several researchers have investigated on the surface morphology and the dimension deviation of the strut of cellular lattice structure. Yan et al. [15] investigated the surface morphology and strut size of SLMed 316L stainless steel gyroid cellular lattice structures with volume fraction of 6%, 8%, 10%, 12%, and 15%. The results show that the fabricated lattice structures exhibited very rough surfaces with many bonded particles and the strut size is larger than target value. Also, the struts size of AlSi10Mg periodic cellular lattice structures fabricated via direct metal laser sintering (DMLS) is also slightly higher than target [15]. Van Bael et al. [16] compared the differences in pore size, strut thickness, porosity, surface area, and structure volume between designed and manufactured cellular Ti-6Al-4V structures. All of the morphologic properties deviated from original design. Leary et al. [17] reported that preferential particle adhesion on the lower surface of lattice structures struts lead to increased roughness. Qiu et al. [18] has carried out a parametric study on the influence of processing conditions on strut structure and compressive properties of AlSi10Mg cellular structure. It revealed that the rough surface, defects, and internal porosity exist due to the violent interaction between the laser beam and pool.

The usage of light metal and lightweight structures are two primary methods to reduce the weight of components [19]. Aluminum alloys are suitable for the purpose. However, it is difficult to produce aluminum alloys by SLM due to its high reflectivity and conductivity [20]. A widely studied aluminum alloy in SLM is cast-alloy AlSi10Mg. Weingarten et al. [21] showed that the AlSi10Mg samples with nearly 100% density can be produced by SLM. Thijs et al. [22] determined that AlSi10Mg components that were fabricated by SLM have an extremely fine microstructure and hence a high hardness when compared to the samples fabricated by conventional approaches.

Although there are many literatures reported the surface quality and dimension accuracy of lattice structure, there are few researches investigated the induced causes in detail. Furthermore, there are no literature investigating the density of the inclined cellular lattice strut and giving a method to obtain high surface quality and dimension accuracy, as well as density simultaneously. This paper investigated the evolution of the molten pool morphology, upper and lower surface roughness and dimensional deviation of SLMed AlSi10Mg inclined struts. The aim of this study is to help to understand the surface roughness and dimensional deviation of SLM process of cellular lattice structure and to obtain a method to achieve high surface quality and dimension accuracy, as well as density simultaneously.

## 2. Experimentals

### 2.1. Materials and SLM Process

In this experiment, powder material of the wrought AlSi10Mg material was supplied by Hengji Powder Technology China (Yueyang, China). The particle size was measured using the Malvern UK Mastersizer 3000 (Malvern Instruments Ltd., Worcestershire, UK). The gas atomized AlSi10Mg powders with spherical shape and particles size in the range of 18–50 μm were used. The composition of powder was measured via ICP-AES (inductively coupled plasma atomic emission spectrometry, PerkinElmer Instruments, Shelton, CT, USA). The chemical composition is shown in Table 1.

**Figure 1:**
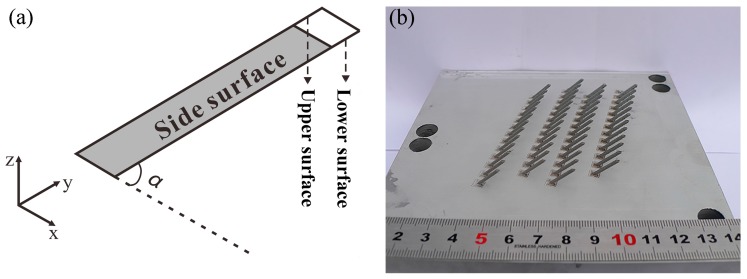
Detail of original CAD model (**a**) and the image of the samples (**b**).

The detail about the origin CAD (Computer Aided Design) model of samples in the experiment is given in Figure 1a. The inclined angle *α* is 45° and the section parallel to substrate is a rectangle with area of 1 mm × $\sqrt{2}\;\mathrm{mm}$. The image of the samples is given in Figure 1b.

The SLM experiments were conducted on a self-developed machine (LSNF-I, Wuhan, China), which is equipped with a continuous wave IPG YLR-200 fiber laser (*λ* = 1.07 μm) with maximum laser power of 200 W and focused spot diameter of about 100 μm. The detailed information about the SLM system was introduced in our previous works [23,24]. Parameters used for preparing the samples in this study are presented in Table 2. A standard alternating x/y raster strategy [25] was chosen for laser scanning paths and the whole fabrication process was carried out in an argon atmosphere. A cleaning system was used to remove the generated fume.

### 2.2. Characterizations

**Figure 2:**
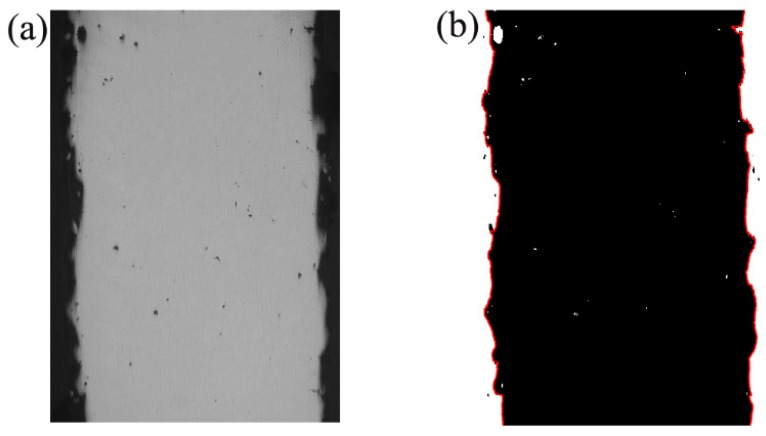
Original image (**a**) and Processed image (**b**).

Prior to characterization, all of the SLMed struts were ultrasonically cleaned in acetone for at least 15 min to remove any trapped loose powder or dirt. The surface morphology was observed by Philips Quanta 200 environment scanning electron microscope (ESEM, Quanta 200, FEI Co. Eindhoven, The Netherlands). After preparation according to the standard metallographic technique, Matlab-based shadow measurements of optical microscope image were used to determine surface roughness and strut size. As shown in Figure 2, the original image is converted to binary one to extract and fit the contour using Matlab R2014 (Mathworks Inc., Natick, MA, USA) to calculate roughness.

**Figure 3:**
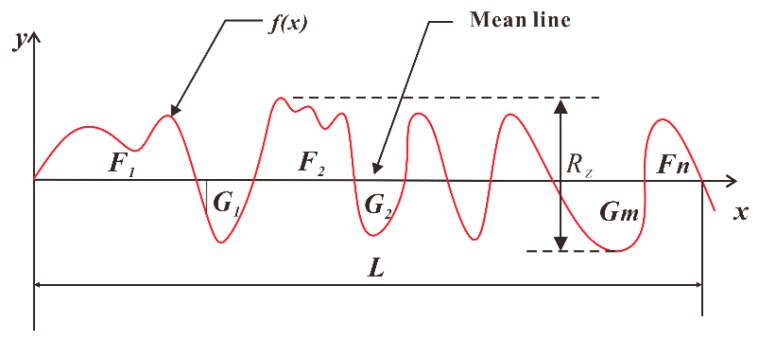
Definition of the parameters $R_{a}$ and $R_{z}$.

As illustrated in Figure 3, $F_{1}+F_{2}+F_{3}+\cdots +F_{n}=G_{1}+G_{2}+G_{3}+\cdots G_{m}$, the average surface roughness $R_{a}$ and peal-to-valley roughness $R_{z}$ can be calculated as follows:

(1) $$R_{a}=\frac{1}{L}\int_{0}^{L}\left|f\left(x\right)\right|dx$$

(2) $$R_{z}=\left|Z_{max}-Z_{min}\right|$$

The strut size is measured as:

(3) $$S_{a}=\frac{\sum_{1}^{N}\left(Pix_{num}\times Pix_{size}\right)}{N}$$

where $S_{a}$ is average strut size, $Pix_{num}$ is the number of pixels between two profiles, $Pix_{size}$ is the pixel size under specific magnification, which shown in Table 3, *N* is the counts of each image. In this paper, *N* is 38 (the 50× metallographic figure of 764 × 1024 pixel and every 20 pixel the strut size was measured once) and the sizes of SLMed struts were determined by mathematically averaging the measurement results of eight images.

**Figure 4:**
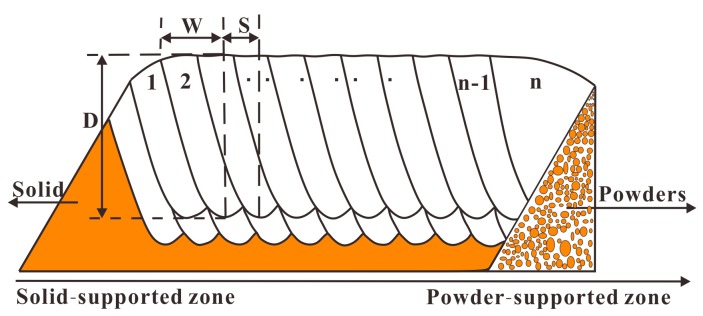
The definition of depth (*D*), half width (*W*), and hatch spacing (*S*) of molten pool, tracks numbers (1, 2 … *n* − 1).

To analyze molten pool, metallurgical samples were chemically etched with Keller’s reagent (containing 95 mL water, 1.0 mL hydrogen fluoride, 1.5 mL hydrochloric acid, and 2.5 mL nitric acid) at room temperature. The Image-Pro plus 6.0 software was used to measure the depth and half width of the molten pool. Figure 4 presents the definition of depth (*D*) and half width (W), tracks number (1, 2 … *n* − 1, *n*). The average depth $D_{av}$ and average half width $W_{av}$ are calculated as Equations (4) and (5), respectively:

(4) $$D_{av}=\frac{1}{n-2}\overset{n-1}{\underset{2}{\sum }}D_{i}$$

(5) $$W_{av}=\frac{1}{n-2}\overset{n-1}{\underset{2}{\sum }}W_{i}$$

where $D_{n},\;W_{n}$ are the depth and half width of the *n*^th^ track, respectively. i=2,3,⋯n−1

## 3. Results and Discussion

### 3.1. Density

**Figure 5:**
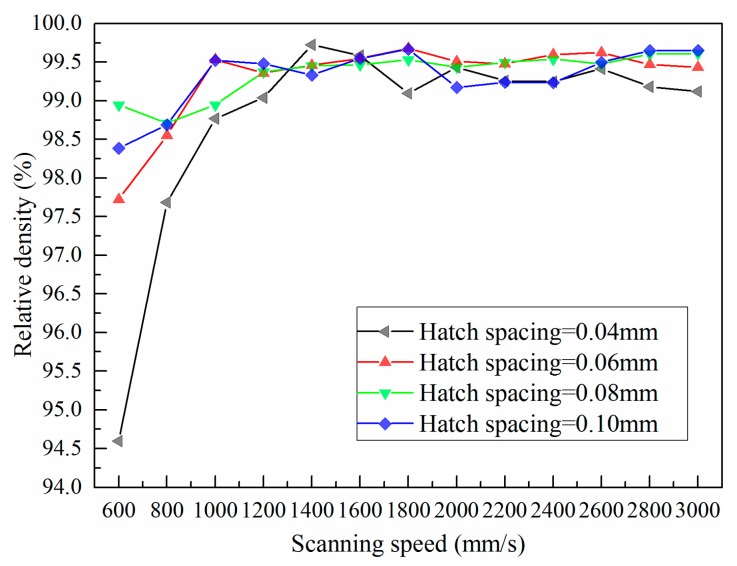
Relative density of SLMed struts.

**Figure 6:**
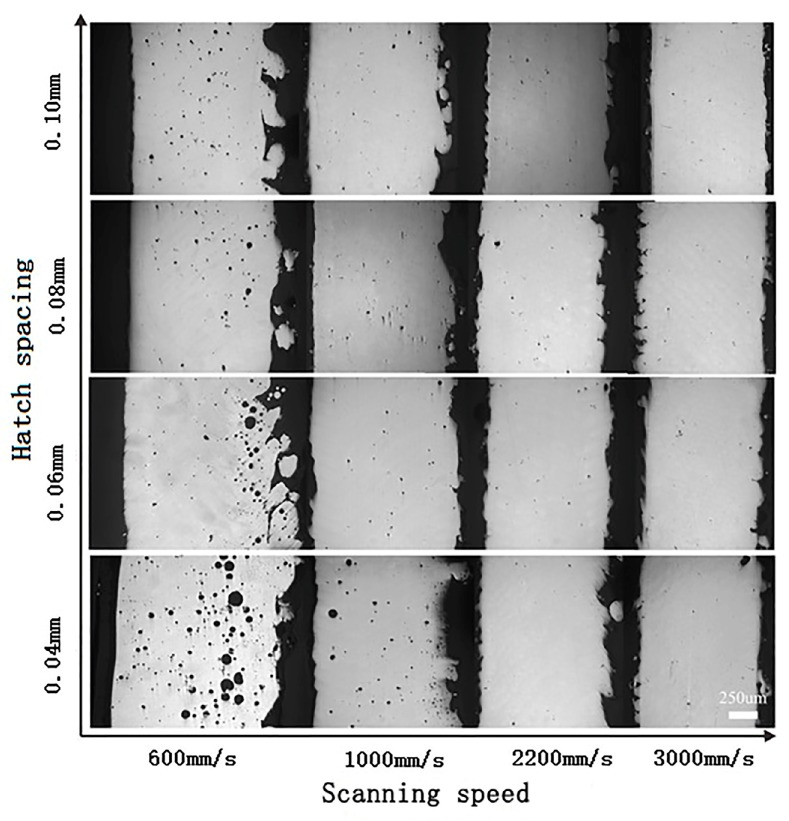
Metallograph of SLMed struts fabricated at different scanning speed and hatch spacing.

Relationship among relative density, scanning speed, and hatch spacing has been investigated. Figure 5 gives the relative density of SLMed struts fabricated at different hatch spacings and scanning speeds. Clearly, the relative density with scanning speed of 600–1000 mm/s are always below 99.0%. Further increasing the scanning speed, the density of SLMed struts is improved effectively. The relative density is always up to 99.0% and not significantly be affected the scanning speed if the scanning speed is in range of 1400–3000 mm/s. Besides, relative density is less dependent on hatch spacing as the scanning speed increasing over 1200 mm/s. Compare with the metallograph of SLMed struts with different hatch spacings and scanning speeds, it can be found that there are many spherical pores in SLMed strut when the hatch spacing and scanning speed are small, e.g., high input energy (Figure 6). The higher the input energy, the higher and larger the pores. More metallic vapor generated due to high energy input. The intensity of liquid metal flowing also leads to the formation of pore. Most of the pores are located at the powder-supported area. When the scanning speed is 3000 mm/s, there are less pores in all of the samples.

### 3.2. Molten Pool Morphology

**Figure 7:**
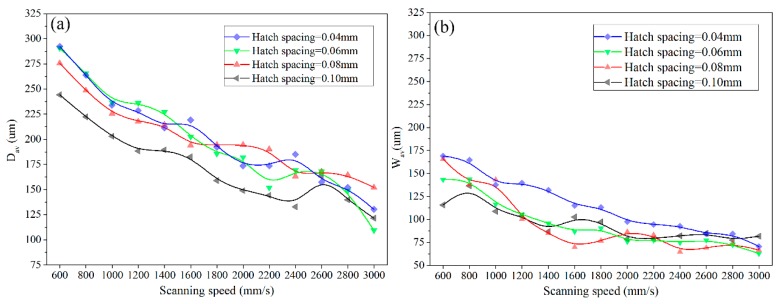
The average depth (**a**) and half width (**b**) of molten pools under different processing parameters.

The average depth and half width of molten pools with different scanning speed and hatch spacing have been studied. Figure 7 shows the variation trend of the measured average depth and half width of molten pools under different processing parameters. Obviously, if the hatch spacing is kept constant, both the average depth and the width of molten pools decrease with the increase of scanning speed. But, the dependence of average half width on scanning speed becomes weak as the scanning speed arrives a definite value. When the scanning speed is kept constant, the molten pool has deeper depth and wider width under smaller hatch spacing.

**Figure 8:**
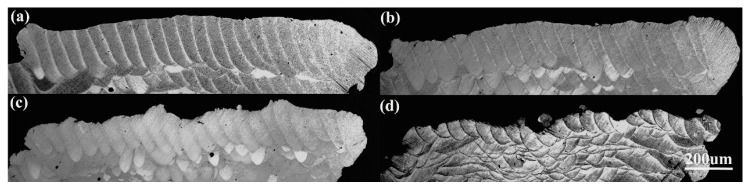
Cross-sectional morphology of molten pool of the struts fabricated by fixed hatch spacing of 0.06 mm and different scanning speed of: (**a**) 1400 mm/s; (**b**) 1800 mm/s; (**c**) 2200 mm/s; and, (**d**) 3000 mm/s.

**Figure 9:**
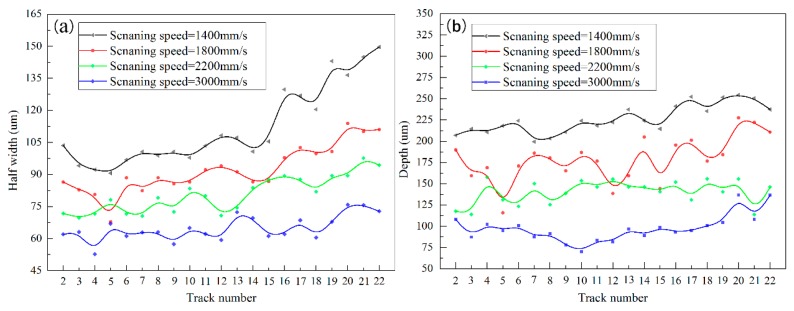
The half width (**a**) and depth (**b**) of molten pool of different tracks.

Accroding to Equations (1) and (2), the *R_a_* and *R_z_* of the samples with different processing parameters have been calculated. Figure 8 shows the cross-sectional morphology of molten pool of the SLMed samples fabricated using different scanning speed at a fixed laser power of 200 W and a hatch spacing of 0.06 mm. The depth and half width of molten pool of different tracks were measured and the results are shown in Figure 9, except for the first and the last track (totally 23 tracks in each layer when the hatch spacing is fixed at 0.06 mm).

It can be seen that half width and depth of the molten pools at the tracks ranging from 18th to 22nd are larger than that of other tracks. As illustrated in Figure 4, all of these tracks are near to the powder-supported zone. For a Gaussian beam with spot diameter of d, the maximum temperature ($T_{max}$) in the center of the beam can be calculated by Equation (6) [26]:

(6) $$T_{max}=\frac{\sqrt{2}AId}{K\sqrt{\pi }}tan^{-1}\frac{\sqrt{2kt}}{d}$$

where A is laser absorptivity coefficient, I is laser intensity, K is thermal conductivity, *k* is thermal diffusivity of molten material, and t is interaction time between laser beam and metal powders. Due to the fully solidified part exhibits higher thermal conductivity than the loose powder [27], the maximum temperature of powder-supported zone $T_{max}^{p}$ is higher than that of solid-supported zone $T_{max}^{s}$, e.g.,

(7) $$T_{max}^{p}>T_{max}^{s}$$

Therefore, the big size molten pool that is generated in powder-supported zone. However, the half width of molten pool changed more significantly than that of the depth. This is due to the fact that the liquid metal has good wettability and flowability at high temperature, which leads to the wide spread. Furthermore, the large difference exists between them when a low scanning speed is applied. This is because that the energy input is large under a low scanning speed, resulting in the significant effect of thermal conductivity.

### 3.3. Surface Roughness

**Figure 10:**
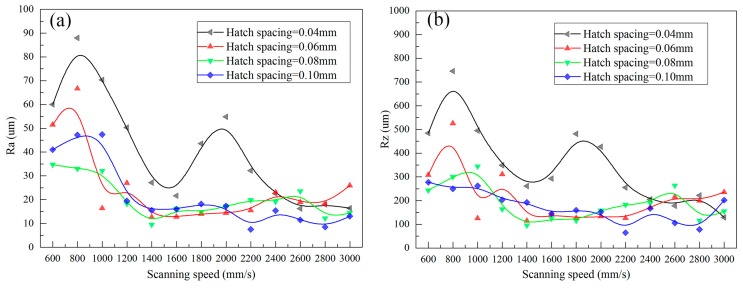
$R_{a}$ (**a**) and $R_{z}$ (**b**) of lower surface.

The arithmetic average roughness ($R_{a}$) and peak-to-valley height roughness ($R_{z}$) of lower surface of SLMed struts that were fabricated by different processing parameters are shown in Figure 10.

**Figure 11:**
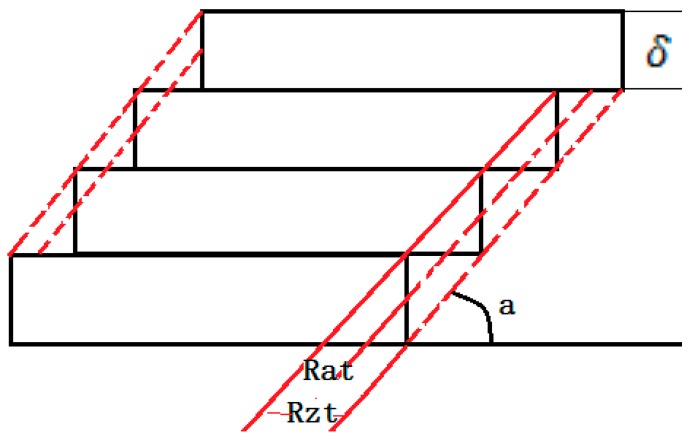
The surface roughness caused by the “stair stepping effect”.

For the strut with inclined angle α, the upper and lower surface roughness caused by the “stair stepping effect”, named as theoretical average roughness $R_{at}$ and peak-to-valley height roughness $R_{Zt}$, as shown in Figure 11, can be calculated from Equations (9) and (10):

(8) $$R_{at}=\frac{1}{L}\int_{0}^{L}\left|fx\right|dx=\frac{1}{4}\delta cos\left(\alpha \right)$$

(9) $$R_{zt}=\delta cos\left(\alpha \right)$$

In this study, $R_{at}$ is about 3.536 μm and $R_{Zt}$ is 14.142 μm. However, from Figure 10, it can be found that $R_{a}$ and $R_{z}$ are much larger than $R_{at}$ and $R_{Zt}$, respectively, indicating other causes that significantly influence the surface roughness. Furthermore, the curves in Figure 10 show a similar trend: when the applied laser power and hatch spacing are fixed, as increasing the scanning speed, $R_{a}$ and $R_{z}$ increase firstly, and then decrease, if the scanning speed is large enough, then the curves turn stable.

**Figure 12:**
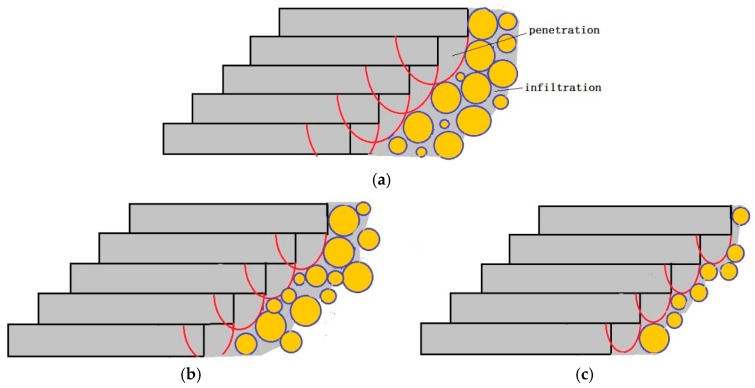
The schematic diagram of lower surface roughness caused by laser penetration and infiltration effect. (**a**) Low scanning speed; (**b**) Middle scanning speed; and, (**c**) High scanning speed.

During SLM, the lower surface roughness is determined by the laser penetration and infiltration effect, as shown Figure 12. The laser penetration has little effect on the surface roughness since the penetration depth is same for same process parameters, e.g., it almost equals to the “stair stepping effect”. However, the infiltration effect influences the surface roughness significantly. If the scanning speed is very low, such as 600 mm/s, the interaction time of *t*, e.g., d/v increases, the temperature of the molten pool center $T_{max}$ also increases, leading to a longer lifetime of molten pools as well as good wettability and flowability. Therefore, more liquid infiltrates into the powder gaps, inducing significant infiltration effect. The liquid fills the gaps very well, inducing plate surface and small surface roughness, as shown in Figure 12a. As the scanning speed increases, the infiltration effect becomes weak, inducing uneven surface and large $R_{a}$ and $R_{z}$, as shown in Figure 12b. Further increasing the scanning speed, the infiltration effect is too weak to bond the powders, $R_{a}$ and $R_{z}$ decreases, as shown in Figure 12c.

There is only little difference in surface roughness as hatch spacing changes, except for the hatch spacing of 0.04 mm. The reason may attribute to that large hatch spacing has little thermal accumulation effect on the adjacent tracks, so the infiltration effect almost same for the same scan speed.

**Figure 13:**
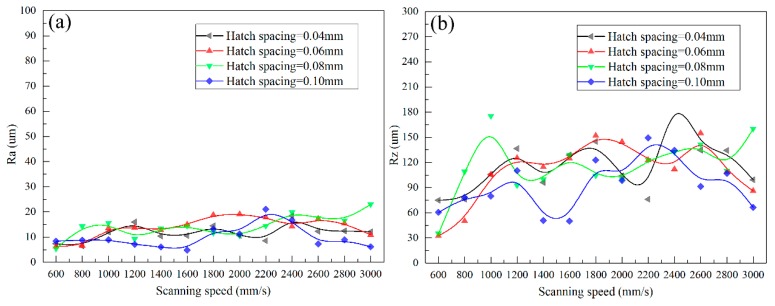
$R_{a}$ (**a**) and $R_{z}$ (**b**) of upper surface.

**Figure 14:**
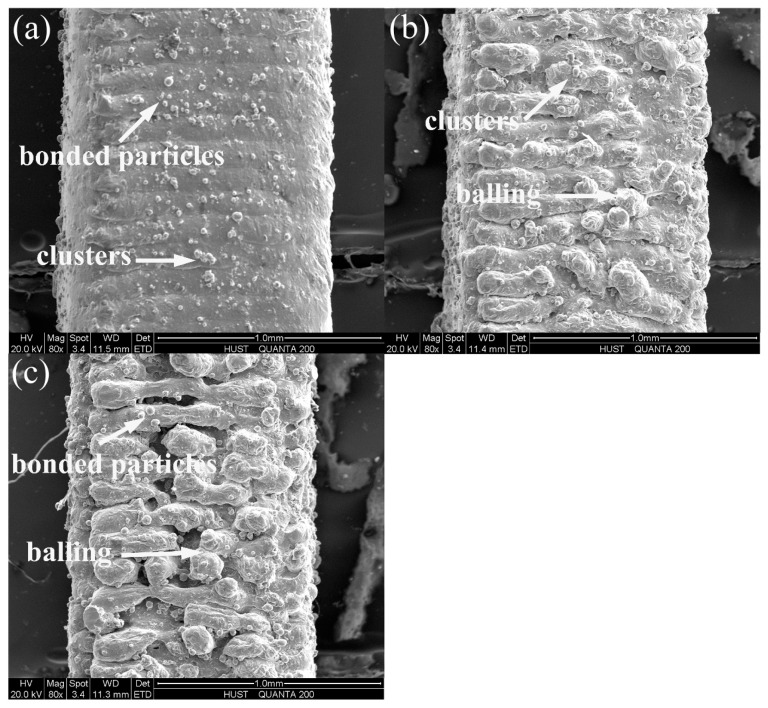
Upper surface morphology of SLMed struts at different scanning speeds: (**a**) 600 mm/s (**b**) 1800 mm/s (**c**) 3000 mm/s (Hatch spacing = 0.08 mm).

Figure 13 gives the variation in upper surface roughness. The upper surface roughness $R_{a}$ and $R_{z}$ are almost constant under different scanning speed and hatch spacing. For the upper surface roughness, “stair stepping effect” is generally considered as the main cause [28]. But, datasets in Figure 13 shows a small deviation from $R_{a}$ and a large deviation from $R_{z}$. This may be attributed to the bonded particles and balling on the stair steps, as shown in Figure 14.

**Figure 15:**
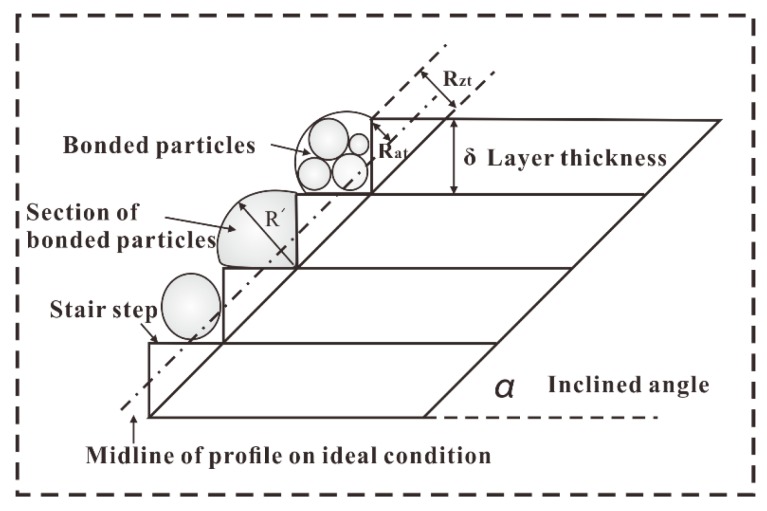
Schematic representation of influence of bonded particles or balling on the profile of upper surface.

As schematically illustrated in Figure 11, $R_{at}$ and $R_{Zt}$ are theoretical average roughness and peak-to-valley height roughness of upper surface on ideal condition, respectively, i.e., no particles or balling on the stair steps. Clearly, bonded particles or balling will change the profile of upper surface. Assuming that the real profile of the surface is denoted as F(x) and the largest value of F(x) is F(u) (as shown in Figure 15). The increment of $R_{a}$ and $R_{z}$ can be calculated as:

(10) $$\Delta R_{a}=\frac{1}{L}\int_{0}^{L}\left|Fx-f\left(x\right)\right|dx=\frac{1}{L}\int_{0}^{L}\left|Fx\right|dx-\frac{1}{4}\delta cos\left(\alpha \right)$$

(11) $$\Delta R_{z}=F\left(u\right)-\delta cos\left(\alpha \right)$$

If $F\left(u\right)>R_{Zt},\Delta R_{a}>0$ and $\Delta R_{z}>0$. In this study, the average powder size (31.4 μm) is about two times of $R_{zt}$, so the bonded particles on the stairs will lead to F(u) is bigger than $R_{Zt}$. The deviator brought by bonded particles cannot be ignored, especially for the starting powders with large particle size. In addition, the balling will become the key factor for the roughness of upper surface under high scanning speeds.

When compared with Figure 10 and Figure 13, the lower surface quality is worse than upper surface, indicating that much more attention should be paid to improve the lower surface quality.

### 3.4. Dimensional Accuracy

**Figure 16:**
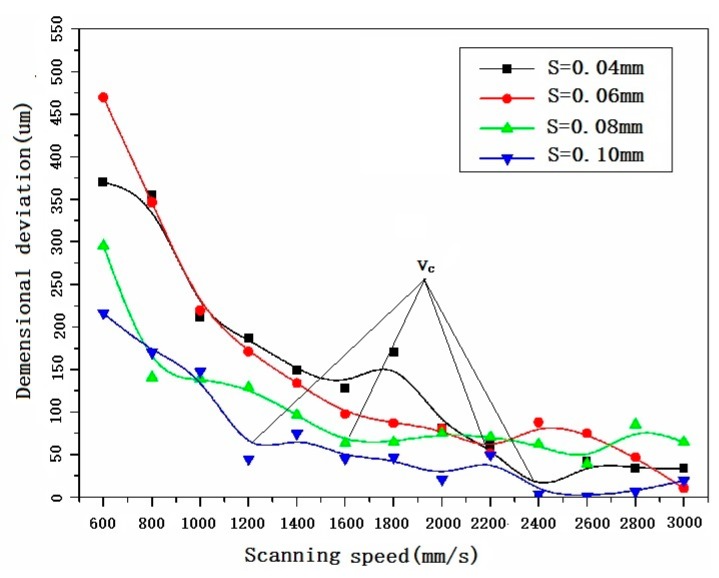
The dimensional deviation of SLMed struts vs. process parameters.

Figure 16 shows the effect of scanning speed and hatch spacing on the dimensional deviation of the SLMed struts. It can be seen that the strut dimensional deviation decreases with the increase of the scanning speed if the hatch spacing is fixed, an inflection point, et al., $v_{c}$, can be found in each curve. If the scanning speed is lower than $v_{c}$, the dimensional deviation decreases rapidly with the increase of the scanning speed. If the scanning speed is larger than $v_{c}$, the dimensional deviation almost keeps constant with the increase of the scanning speed. The smaller the hatch spacing, the larger the $v_{c}$. When comparing Figure 16 with Figure 7b and Figure 10, it can be found that they have similar shape, suggesting that the width of molten pool and surface quality are the key factors that leading to size deviation of SLMed samples. In order to clarify the relationship among molten pool morphology, upper and lower surface quality, and dimensional accuracy, Pearson’s Correlation coefficient analysis was applied. The $R_{a}$ is taken to represent the surface quality. Dimensional deviation $S_{a}$, $R_{a}$ and average width of molten pool d are calculated as follows:

(12) $$\Delta S_{a}=S_{a}-0\;\Delta W_{av}=W_{av}-d\;\Delta R_{a}=R_{a}-R_{at}$$

The calculated Pearson’s correlations were presented in Table 4.

Clearly dimensional accuracy has strong correlation with lower surface quality and width of molten pool. A strut with small molten pool and good surface quality has high dimensional accuracy. According to the analysis, high scanning speed can satisfy the requirements. Therefore, high scanning speed should be applied to decrease the dimensional deviation when the inclined strut is fabricated.

## 4. Conclusions

Selective laser melting of AlSi10Mg inclined struts has been carried out in this study. The influence of scanning speed and hatch spacing on the molten pool evolution, upper and lower warding surface roughness, as well as the dimensional deviation of the SLMed samples was investigated systemically. Based the investigations, the following conclusions can be drawn as follows:

(1) Both the average depth and half width of molten pool decrease as the scanning speed increases. But, the dependence of the half width becomes weak when the scanning speed is high enough.
(2) Bigger molten pool is formed and the half width changed greatly than that of the depth when laser irradiates from solid-supported zone to powder-supported zone.
(3) The lower surface quality is deteriorated under low scanning speed due to infiltration effect. The upper surface roughness has small fluctuation under different hatch spacing and scanning speed due to the bonded particles and balling on stair step. The surface roughness of lower is larger than that of upper.
(4) Dimensional accuracy has strong correlation with lower surface quality and width of molten pool. The dimensional accuracy can be improved by applying high scanning speed when the inclined strut was fabricated.
(5) SLMed AlSi10Mg inclined strut with low surface roughness and high dimensional accuracy as well as high density can be achieved simultaneously by using high scanning speed.

## Figures and Tables

**Table 1:** The chemical composition of the AlSi10Mg powders.

Element	Al	Si	Mg	Fe	Ti	Ni	Cu	Zn	Pb	Sn	Mn
Wt %	Balance	10.04	0.47	1.42	0.018	0.043	<0.01	<0.01	<0.01	<0.01	<0.01

**Table 2:** Processing parameters used in this study.

Processing Parameters	Value
Laser power P,W	200
Scanning speed v, mm/s	600–3000
Layer thickness δ,μm	20
Hatch spacing S, mm	0.04−0.10

**Table 3:** Pixel size under specific magnification.

Magnification	um/Pixel (*x* Axis)	um/Pixel (*y* Axis)
50×	1.92926	1.92926
100×	0.4761905	0.4746836

**Table 4:** Pearson’s Correlation coefficient.

Pearson’s Correlation	$\Delta R_{a}$ of Upper Surface	$\Delta R_{a}$ of Lower Surface	$\Delta W_{av}$	$\Delta S_{a}$
$\Delta S_{a}$	−0.03063976	0.7003351	0.7395423	1
